# Efficacy of Selective Grinding Guided by an Occlusal Splint in Management of Myofascial Pain: A Prospective Clinical Trial

**DOI:** 10.2174/1874210601711010301

**Published:** 2017-06-30

**Authors:** Felipe J. Fernández-González, Jorge Cabero-López, Aritza Brizuela, Ivan Suazo, Esteban Pérez-Pevida, Teresa Cobo, Oier Montalban, Markel Diéguez-Pereira, David Chávarri-Prado, Iker Bellanco de la Pinta, Antonio Jiménez-Garrudo

**Affiliations:** 1 Department of Surgery and Medical-Surgical Specialties, Medical and Dental School, University of Oviedo, Instituto Asturiano de Odontologia, Oviedo, Spain; 2 Facultad de Ciencias de la Salud, Universidad Autónoma de Chile, Chile; 3 Faculty of Medicine and Health Sciences, University of Oviedo, Oviedo, Spain; 5 Department of Surgery, Gynecology and Obstetrics, Faculty of Sports and Health Sciences, University of Zaragoza, Huesca, Spain; 6 Director de Postgrado e investigación, Universidad Autónoma de Chile, Chile; 7 Faculty of Medicine and Health Sciences, University of Salamanca, Salamanca, Spain; 8 Department of Stomatology I, Faculty of Medicine and Dentistry, University of the Basque Country, Leioa, Spain.; 9 Department of Surgery, Faculty of Medicine, University of Salamanca, Salamanca, Spain

**Keywords:** Occlusion, Selective grinding, Temporomandibular disorder, Occlusal splint

## Abstract

**Background:**

For patients whose centric relation (CR) has not been considered at the start and during treatment, the task of achieving an occlusal scheme that works together with the temporomandibular joint, the muscles, and the structures of the stomatognathic apparatus becomes a major concern.

**Objective:**

This study aims to describe a reproducible, predictable and to date unreported procedure of selective grinding guided by an occlusal splint and to analyze condylar position (CP) based on the skeletal pattern.

**Methods:**

A total of 72 symptomatic patients (38 females and 34 males) were classified into three groups: hyperdivergent, intermediate and hypodivergent. CP was quantified by mounted casts on a measures condyle displacement (MCD) device. Helkimo index was also performed in order to assess the severity of the temporomandibular joint (TMJ) disorders attending to clinical dysfunction, occlusal state and anamnestic dysfunction. Once the stability had been obtained, the splint was progressively reduced until the maximum intercuspation (MIC) was achieved.

**Results:**

The vertical displacement was found to be significantly different between the hyperdivergent and other two groups (*p*<0.01). Comparisons of MCD analysis before and after the selective grinding procedure identified a statistically significant difference in the horizontal and vertical CP (*p*<0.01) between the different groups whereas the Helkimo Index showed a clear improvement of TMJ disorders.

**Conclusion:**

All facial types, specially the hyperdivergent face type, showed a reduction in condylar displacement (CD) and less craniomandibular symptoms using this procedure, making it an excellent technique for clinicians.

## INTRODUCTION

When approaching oral rehabilitation, clinicians can refer to two established positions of the mandible to guide treatment planning: centric relation (CR) and maximum intercuspation (MIC) [1].

MIC is defined as the complete intercuspation of the upper and lower teeth independent of condylar position. Specifically, the jaw position and its relationship with the rest of the cranial structures are dictated by the teeth.

The centric relation (CR) is a maxillomandibular relationship, where the condyles are centered transversely by coordinated masticatory muscles and are articulated by the medial portion of their respective discs, with the disc-condyle complex in an antero-superior position against the surface of the articular eminence [2].

This craniomandibular relationship has been described as the most stable and comfortable position of the mandible in which the joints can be loaded without discomfort [2].

Many authors have highlighted the differences between these two mentioned positions, which do not usually coincide in the general population; this means that a shift from the CR position is required in order to achieve the MIC position [3-6].

Okeson argued that this lack of harmony between the musculoskeletal stable position of the condyles and the intercuspal position of the teeth represents an orthopedic instability [7]. CR-MIC discrepancies can lead to increased joint space and loosening of the tight relationship between the condyle and the cranial base described above. This contributes to articular instability and the potential development of temporomandibular disorders (TMD) [8]. Several studies have shown [9 , 10] that the clinical relevance of this situation increases when this shift is greater than 2 mm in either the horizontal or vertical axis.

Because CR is the most consistent and reproducible positional reference [11-14], accurate studies of dental and maxillomandibular relationships are dependent on the assessment of CR. For this reason, a set of mandibular position indicators have been developed in order to meet the need to accurately identify and measure condylar displacement in three dimensions [15].

When orthodontic treatment is finished, the clinician should ensure that the criteria for optimal orthopedic stability in the masticatory system are fulfilled. This involves ensuring the simultaneous contact of all possible teeth when the condyles are in their most upper-anterior position and resting against the posterior slopes of the articular eminences, with the discs properly interposed [7]. Procedures such as selective grinding [16-18] can facilitate this ‘no shift’ situation. Selective grinding of the tooth surfaces to produce occlusal adjustment has been described as a valid adjunct to orthodontic treatment for improving the overall contact pattern of the teeth [19 , 20]. However, the procedure must be well planned, based on precise diagnosis, and use a controlled and accurate method.

For patients whose CR has not been considered at the start and during treatment, the task of achieving an occlusal scheme that works together with the temporomandibular joint, the muscles, and the structures of the stomatognathic apparatus becomes a major concern, especially in patients with hyperdivergent skeletal patterns, who usually present with even larger discrepancies [12 , 21].

The aim of this study was to describe a reproducible, predictable and to date unreported procedure for selective grinding with an occlusal splint applied to produce a discrepancy-free MIC-CR situation in patients treated orthodontically without initial CR assessment. Furthermore, we wanted to confirm the differences in the magnitude of the shifts produced in relation to the patients’ skeletal pattern.

## MATERIALS AND METHODS

The study protocol was reviewed and approved by the León Hospital Ethics Committee, Spain (#1586). The study was performed in accordance with Spanish laws and the Declaration of Helsinki II for research involving human subjects.

**Fig. (1):**
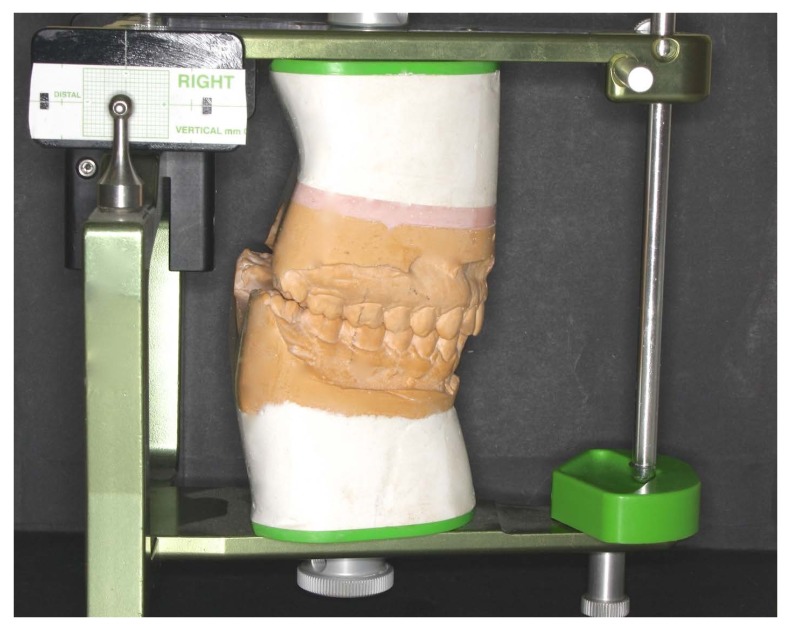
Study casts mounted in centric relation on a Ad2 measures condyle displacement device.

**Fig. (2):**
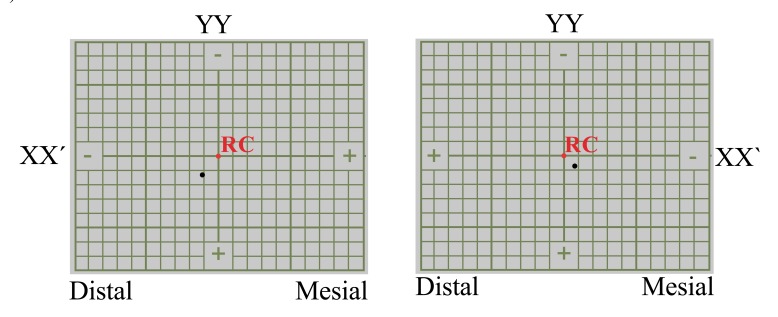
A mandibular position indicator registration. The red and black dots represent the centric relation and maximum intercuspation, respectively. The axial displacements are as follows: for the right condyle, -0.9 mm in XX' and +1.1 mm in YY'; and for the left condyle, -0.9 mm in XX' and +0.8 mm in YY'.

The sample population was comprised of 72 Caucasian, symptomatic individuals who had completed orthodontic treatment at least five years previously (38 females and 34 males; mean age of 23.2 years). The data were obtained from the following sources: medical records and clinical examinations; lateral cephalograms in MIC; casts taken in CR mounted on a semi-adjustable articulator (AD ^2^^®^; Advanced Dental Designs Inc, Riverside, USA); and condylar position studies using the ‘Measures Condyle Displacement’ device (MCD) (AD^2®^; Advanced Dental Designs Inc, Riverside, USA), which measures the three-dimensional position of the condyle in the maximum intercuspal position (MIP) (Figs. **1** & **2**).

**Fig. (3):**
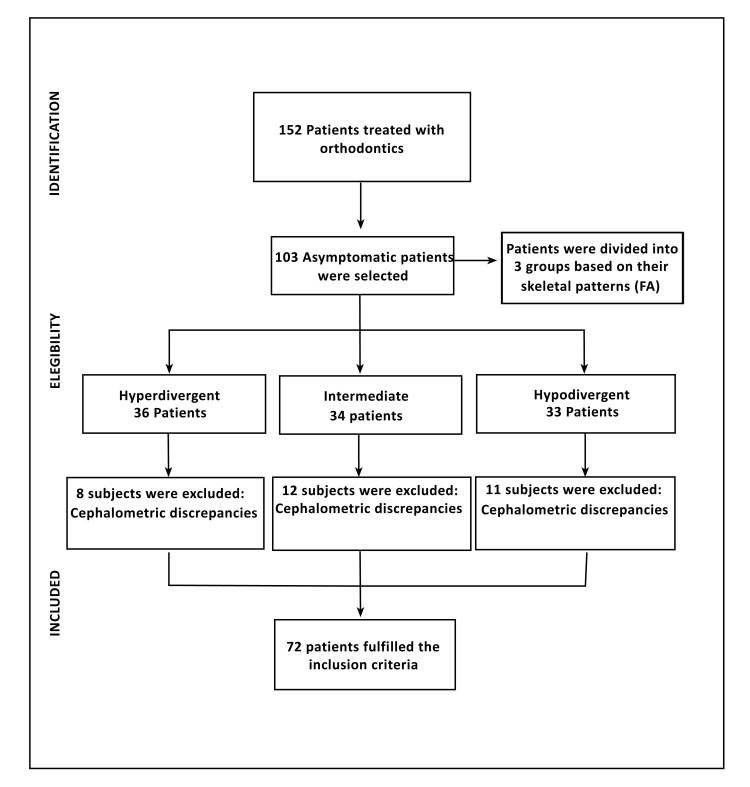
Flow chart showing the subject inclusion process.

The patients were recruited from a private orthodontic practice (Orthodontic Aesthetics Centre, León, Spain) and selected for inclusion on the basis of a clinical examination using the predetermined inclusion criteria shown in (Fig. **3**).

The participants were divided into three groups according to their facial pattern, which were classified on the basis of the facial axis (FA; Basion-nasion/pterygoid-gnathion) according to Ricketts cephalometric analysis [22]. Patients with an FA ≤ 86º were classified as hyperdivergent, patients with an FA ≥ 94º were classified as hypodivergent, and the remaining patients (FA: 87º-93º) were classified as intermediate.

The cephalometric analysis was performed by two experienced orthodontists. Patients for whom the cephalometric results did not coincide were excluded.

A single operator (J.P.F) with over 20 years of experience registered all the clinical and laboratory data. An arbitrary face bow and an articulator (AD^2®^; Advanced Dental Designs Inc, Riverside, USA) were used for mounting the stone casts (Ade Stone, Whip Mix Corp., Louisville, KY) produced from CR wax records (Delar Bite Registration Wax; Delar^®^ Corporation, Lake Oswego, OR, USA). The horizontal and vertical CP was evaluated using a single MCD and MIC wax record (Moyco^2®^ Industries Inc., Philadelphia, PA, USA). Helkimo Index was also performed at this stage (T0) in order to evaluate the patients´clinical condition.

The MIC records were obtained before CR registration by asking the patient to bite firmly on softened wax. After chilling the wax register in cold water to harden it, the accuracy of the record was checked in the mouth. The Roth power centric technique [23] was used to register the CR immediately after neuromuscular deprogramming with the patient relaxed and reclined at 45º. An anterior acrylic device was interposed between the dental arches for 25 min. CR bite registration was performed in two stages. The patient bit into softened wax to register the anterior section, guiding the mandible during closure in order to avoid protrusion; the cusps responsible for premature inter-arch contact were kept 1-mm apart.

Afterwards, the wax register was hardened in cold water and then interposed between the arches simultaneously with a second softened wax section to register the posterior region. The mandible was guided during closure, and when the anterior teeth were brought into fit with the corresponding anterior wax indentations, the patient was asked to bite firmly. As the posterior wax section was softened, muscular strength helped to adjust the vertical intra-articular condylar position [23].

A micrometer (Series 293 MDC-MX Lite; Mitutoyo U.S.A^®^, Illinois, U.S.A.) was used to measure the horizontal (XX') and vertical (YY') CD components, and each measurement was repeated five times. The average of the two closest values was taken as the definitive value.

Following the recommendations made by Joao Ponces *et al*. [21], the same operator carried out the CR registration, cast mounting procedures, and MCD measurements over two sessions within a 10-d period in order to evaluate intra-operator error and test the reproducibility of CR registration. For these procedures, eight patients were randomly selected and the clinical measurements were obtained twice during each session. During the second session, two new CR records were used to remount the mandibular cast to each previous articulator mounting. This procedure was repeated twice for each mounting set. The new sets of MIC records allowed for comparisons of the MCD registrations. The results were systematized into tables for analysis.

**Fig. (4):**
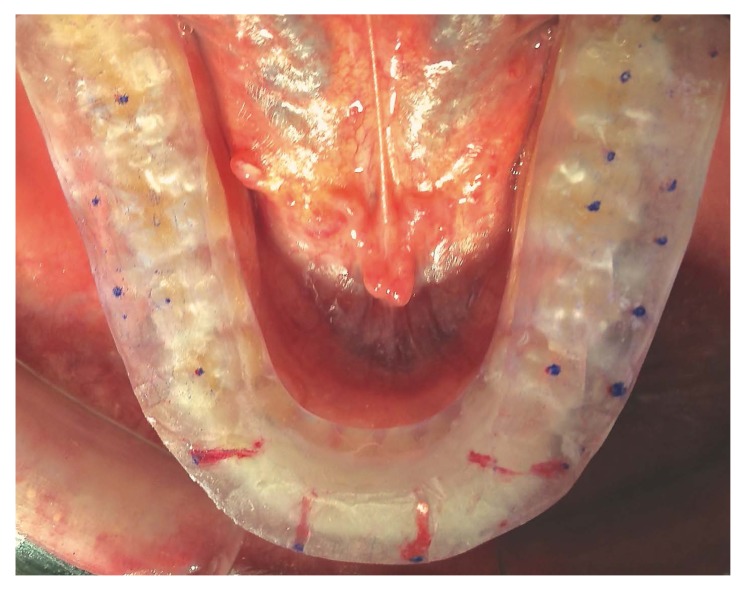
Occlusal adjustment with the splint positioned on the teeth.

An occlusal, acrylic resin splint in CR was built and placed on the mandibular arch of each patient. This splint was fabricated in the articulator with 3 mm clearance from the first dental contact in CR. Afterwards, initial trimming in the articulator allowed to obtain centric stops and a mutually protected occlusal pattern. A single experienced operator adjusted each splint every two weeks during three months in order to obtain a mutually protected occlusion until stabilization was reached (Fig. **4**).

**Fig. (5):**
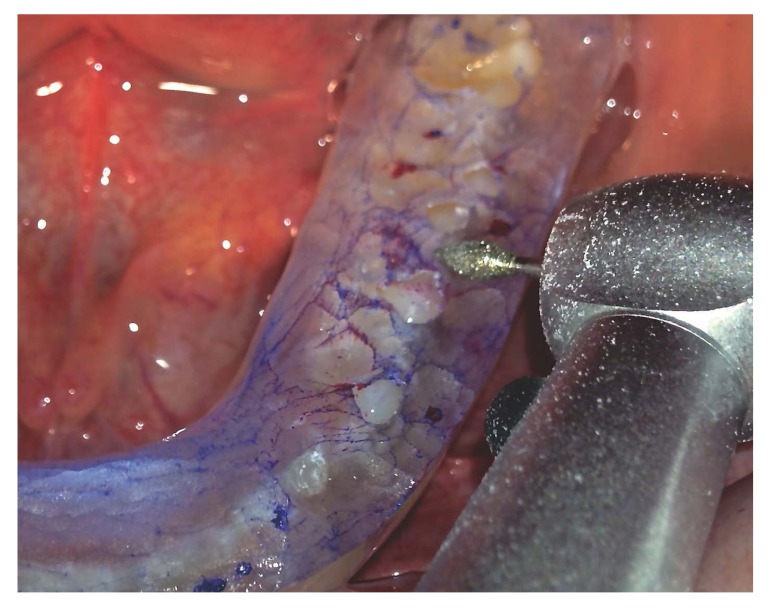
The selective grinding process guided by an occlusal splint.

Then, a new Helkimo index(T1) and MCD study were performed. When the latter test showed that the condyles were close to the CR and the patient continued without symptoms, this indicated that the MIC had changed and showed a clear premature contact. Then, the occlusal splint was ground down to achieve the correct occlusal adjustment (Fig. **5**).

Red and blue 12 µm occlusion foil (Hanel^®^, Langenau, Germany) was used to identify tooth contact, and the teeth were ground down with an ultra-fine diamond bur (Fig. **5**). The procedure was repeated as many times as necessary until the splint was completely perforated, and the patient occluded in MIP without any interceptive occlusal contact.

An additional MCD and Helkimo Index (T2) were performed five months after selective grinding to check the final CP.

### Statistical Analysis

Statistically significant differences between groups were identified using the Student t-test. Direct measurements error was analyzed using an analysis of variance (ANOVA). The error propagation equation was applied to analyze the indirect measurements.

## RESULTS

Analysis of both the direct and indirect measurements error showed an error margin below 4%, which is considered acceptable according to previous research [24].

The horizontal displacement values ranged from -2.8 mm to 3.72 mm, while the vertical displacement values ranged from 0 to 3.82 mm (Table **1**).

No negative vertical CD (YY < 0) data were observed in the total sample (n=144 condyles); the horizontal displacement CD-XX' ≥ 2 mm and CD-XX' ≤ -2 mm occurred in 10/144 condyles (6.94%), while vertical displacement CD-YY ≥ 2 mm occurred in 36/144 condyles (25%). CD-XX' was more frequent in the hypodivergent group (6.64%). Along the YY axis, the frequency of CD-YY ≥ 2 mm was higher in all groups, with values of 36.22%, 20.24%, and 28.32% in the hyperdivergent, hypodivergent, and intermediate groups, respectively. All patients showed a deviation between the MIC and CR, presenting premature contacts in their dynamic occlusion. Table **2** shows the MCD readings just after the selective grinding procedure. No horizontal displacement CD-XX' ≥ 2 mm or CD-XX' ≤ -2 mm occurred in any case (0%), whereas vertical displacement CD-YY' ≥ 2 mm occurred in 5/144 condyles (3.47%). CD-XX' was observed more frequently in the hypodivergent group (4.24%). Along the YY' axis, the frequency of CD-YY was much lower in all cases compared to before the procedure, although in the hyperdivergent group, 7.5% of the condyles registered a CD ≥ 2 mm. The values of the horizontal displacement ranged from -0.52 mm to 1.16 mm, whereas the values of the vertical displacement ranged from 0 to 2.48 mm (Table **2**).

The results of the t-test showed that before the selective grinding procedure, vertical displacement was significantly different between the hyperdivergent and hypodivergent groups (*p*<0.001) and the hyperdivergent and intermediate groups (*p*<0.001). Comparison of MCD analysis before and after the selective grinding procedure found statistically significant differences in the horizontal and vertical CD (*p*<0.001) (Table **3**). All the results remained stable, and patients remained asymptomatic 5 months after the selective grinding procedure and subsequent MCD analysis (Table **3**).

Helkimo Indexes results (Table **4**) showed an improvement of each patient group attending to clinical dysfunction, occlusal state and anamnestic dysfunction, not only before wearing the splint (T1) but also five months after the grinding (T2). Comparing the results before and after the splint was prescribed the three groups referred an improvement in every aspect of the test, whereas all the results showed a clear enhancement 5 months after the selective grinding procedure.

## DISCUSSION

When orthodontic fixed appliances are removed, clinicians should check the occlusion obtained at the end of treatment. At this time, some adjustment may be required to achieve a correct final occlusion. Yet, the precise requirements of that occlusal correction and the role CR should play still evokes passionately opposed opinions [25-32]. Moreover, some authors [28] have pointed out there is no benefit of seeking a no CR-MIC discrepancy though this idea is far from building consensus as other studies claim that CR is a clinically, repeatable jaw position that should be of reference when dealing with oral rehabilitation or orthodontic treatment [26, 29-31]Therefore, it could be inferred there should ideally be a ‘no shift’ CR-MIC situation that constitutes a condition of ideal biomechanical and functional occlusion. Okeson [32] describes that ideal situation as “simultaneous contacts of all possible teeth with both condyles in their most superoanterior position, resting the posterior slopes of the articular eminences,” which is a widely studied comfort situation for all the elements that play a role in the stomatognathic system. This situation benefits not only the temporomandibular joint and neuromusculature but also the periodontal tissues [17]. Obtaining this equilibrium requires a process that involves a clockwise rotation of the mandible and relies on detecting key factors such as fulcrum points (first contact point), especially in vertical pattern patients. However, the question remains as to how the experienced clinician should detect these fine occlusal details?.

The rationale behind the present study was to address the clinical need for a precise, safe, and well-assessed procedure for obtaining the desired equilibrium described above. In addition, we aimed to review the few studies available on the relationship between skeletal patterns and CR-MIC discrepancies. To meet this clinical need, the present study introduces a novel and unreported procedure to detect and correct occlusal interferences and premature contacts, while at the same time seeking orthopedic stability in the masticatory structures, in orthodontic patients treated without earlier CR assessment. In this context, this study also aimed to determine which patients, in terms of their skeletal patterns and CR-MIC discrepancy, are more likely to benefit from this procedure.

One limitation of this study is that no deprogramming technique was applied before CR registration to assess condylar displacement, which may have biased the results. Nevertheless, patients wore the centric-designed occlusal splints around the clock, which should have avoided errors resulting from the lack of a deprogramming technique [33].

The initial MCD analysis performed at the beginning of the present study showed a discrepancy between CR-MIC positions of over 2 mm in 36/ 120 condyles. Many authors have recognized a 2 mm difference as clinically significant [9, 10], with clinical implications including signs and symptoms of TMD. The present findings corroborate the results obtained by Crawford [10] and Young [9] in similar studies, which analyzed the frequency of discrepancies greater than 2 mm. The study also observed a greater magnitude in the vertical plane in all groups. The MCD results exhibited a horizontal displacement (CD-XX' ≥ 2 mm or CD-XX' ≤ -2 mm) in 6/120 condyles (5%), whereas vertical displacement (CD-YY ≥ 2 mm) occurred in 26.66% (32/120) of the condyles. These findings agree with the finding of Hidaka *et al* [30]. Similarly, Ponces *et al*. [21] analyzed 216 condyles and found almost identical results to the present study, with a CD ≥ 2 mm in 27.77% of the cases in the vertical plane and a CD ≥ 2 mm or CD ≤ 2 mm in 6.49% in the horizontal plane. Nevertheless, other studies [33] have found conflicting results, suggesting that some factors related to the influence of the skeletal pattern on CR-MIC discrepancies remain unexplained. For this reason, further studies that take the transverse dimension into consideration are needed before firm conclusions can be drawn.

Regarding facial pattern and its relationship with condylar displacement, the results of the present study agree with previous research carried out by Ponces *et al*. [21] and Girardot [34], who showed a greater CD along the vertical axis in hyperdivergent patients in comparison with the hypodivergent and intermediate patients.

In the present study, horizontal displacement was more frequently observed in the hypodivergent group (6.64%). This result differs from data obtained by Girardot [34], who found greater displacement in the hyperdivergent group, which included only extreme pattern patients. Nevertheless, the findings by Ponces *et al*. [21]support the present findings and obtained greater horizontal displacement among the hypodivergent patients compared with the hyperdivergent and intermediate groups, although the differences did not reach statistical significance. Along the vertical axis, the frequency of CD-YY ≥2 mm was higher in all groups, with registered values of 36.22%, 20.24%, and 28.32%, in the hyperdivergent, hypodivergent, and intermediate groups, respectively. This higher frequency among the hyperdivergent patients compared with the other two groups is consistent with the results reported by Ponces *et al*. [21], who described vertical displacement in 34.72% of hyperdivergent patients. In both studies, the results were higher than those reported by Girardot [34].

In the hyperdivergent, hypodivergent, and intermediate groups, the magnitude of means (absolute values) along the horizontal axis (0.82 mm, 0.97 mm, and 0.63 mm) and the vertical axis (1.85 mm, 1.18 mm, and 1.13 mm) are closely related with those published by Ponces *et al* [21]. Interestingly, both studies obtained similar results, in contrast with the lower discrepancies reported by Girardot [34]. However, Girardot’s [34]study has a number of differences from the present work including the criteria used for sample selection, the neuromuscular deprogramming methodology, the CR recording techniques, and most importantly, the lack of an intermediate group, which could explain the lower results in the hypodivergent patients and the higher results in the hyperdivergent patients.

When studying MCD, the literature points to greater condylar displacement in the vertical axis and hyperdivergent patients are susceptible to greater discrepancies.

After 3 months of wearing the occlusal splint and making the corresponding adjustments every 2 weeks in order to establish a mutually protected occlusal scheme, stabilization was achieved and subsequent MCD studies showed almost complete correction of CR-MIC occlusal discrepancies and an altered MIC position, which was evidenced by premature contacts. The Helkimo index carried out at this point also showed decreased values in each group of patients, similarly to the study of Nascimento *et al.* [35] analyzing the correlation of the employment of occlusal splints for a similar period of time and the Helkimo Index results. Thus, once an optimal condyle position has been reached, the clinician should perform accurate adjustments in order to reach a situation free of MIC-CR discrepancies. To do this, several methods of occlusal adjustment have been described for treating TMD. Christensen *et al*. [16] recommended occlusal equilibration only after an adequate observation period and reduction in muscle pain. Furthermore, Wenneberg *et al*. [17] proposed the combined use of a full occlusal splint and occlusal adjustment. The selective grinding procedure is an irreversible process for reshaping teeth by accurately modifying specific areas of the crown according to a previously established pattern, in order to remove interferences and premature contacts. In the present study, once the condyles were seated, the use of the splint identified these premature contacts in MIC.

After splint-guided selective grinding had been performed, CR-MIC discrepancy analysis showed statistically significant differences in both the vertical and horizontal axis in all three groups. Moreover, the final MCD analysis carried out five months after selective grinding found no clinical differences, suggesting that short- to medium-term musculoskeletal stability had been achieved. This fact was endorsed by the Helkimo Index performed at the same point, similarly to other studies in which an occlusal adjustment carried out in patients with chronic myofascial pain [36] or temporomandibular dysfunction [37] resulted in a significant reduction of the EMG activity of the chewing muscles, patients´self-reported pain or patients´ TMD symptoms.

## CONCLUSION

Patients who have completed orthodontic treatment, especially those with hyperdivergent facial patterns or symptomatic individuals, will benefit from a means of achieving a centric relation position with no MIC discrepancies, chiefly if they present TMJ disorders. In this context, selective grinding guided by an occlusal splint is one such procedure, and the present study observed a reduction in vertical CD in all groups. This predictable and reproducible method produced decreases in final discrepancies and led to a greatly improved occlusal framework and the improvement of TMJ disorders. This procedure offers the clinician a safe, accurate, and inherently simple procedure for selective grinding. As the study only monitored the patients for 5 months, further research analyzing the long-term outcomes of the procedure are needed to confirm the present findings.

## Figures and Tables

**Table 1:** The initial MCD analysis expressed as the mean values (SD), minimum and maximum (mm) and horizontal (XX`) and vertical (YY) CD in the different facial pattern groups.

Facial Pattern/CD	Mean (SD) XX`	YY		Minimum (mm) XX	YY		Maximum(mm) XX	YY	
Hyperdivergent (n=56)	0.82 (0.67)	1.85(0.92)	-2.26		0		3.72		3.82
Hypodivergent (n=44)	0.97 (0.72)	1.18(0.98)	-1.92		0		3.5		3.2
Intermediate (n=44)	0.63 (0.83)	1.13(0.96)	-2,8		0		3.32		3.26

XX` Means are expressed as absolute values

SD: standard deviation; CD: condylar displacement.

**Table 2:** Comparison of the MCD analysis before and after selective grinding guided by an occlusal splint. The mean values (SD), minimum and maximum (mm) and horizontal (XX`) and vertical (YY) CD in the different facial patterns groups.

Facial Pattern/CD	Mean (SD) XX	YY	Minimum (mm) XX	YY	Maximum(mm) XX	YY
Before selective grinding						
Hyperdivergent	0.20 (0.41)***	0.31 (0.45)***	-0.42	0	1.11	2.38
Hypodivergent	0.23 (0,55)***	0.22 (0.62)***	-0.45	0	0.92	0.31
Intermediate	0.15 (0.51)***	0.17 (0.78)***	-0.38	0	1.11	0.17
After selective grinding						
Hyperdivergent	0.22 (0.32)***	0.35 (0.45)***	-0.46	0	1.16	2.48
Hypodivergent	0.27 (0,45)***	0.28 (0.62)***	-0.52	0	0.98	0.36
Intermediate	0.18 (0.83)***	0.19 (0.78)***	-0.28	0	1.12	0.22

XX` Means are expressed in absolute values SD: standard deviation; CD: condylar displacement. ***p<0.01

**Table 3:** Comparison of the initial MCD analysis and the MCD analysis after five months of selective grinding.

Facial Pattern/CD	MCDi Mean (SD) XX	YY	MCDl Mean (SD) XX	YY
Hyperdivergent	0.82 (0.67)	1.85 (0.92)	0.28 (0.42)***	0.33 (0.38)***
Hypodivergent	0.97 (0.72)	1.08 (0.98)	0.31 (0,56)***	0.32 (0.46)***
Intermediate	0.63 (0.83)	1.19 (0.96)	0.22 (0.91)***	0.26 (0.54)***

XX` Means are expressed in absolute values

SD: standard deviation; CD: condylar displacement; MCDi: Initial measures condyle displacement; MCDl: Last measures condyle displacement.

***p<0.01

**Table 4:** Helkimo Index of the initial analysis (T0), before wearing out the splint (T1) and after five months of selective grinding (T2).

**Clinical Dysfunction Index**									
	**Hyperdivergent(%)**			**Intermediate(%)**			**Hypodivergent(%)**		
**T0**	**T1**	**T2**	**T0**	**T1**	**T2**	**T0**	**T1**	**T2**
clinically symptom-free	15	68	85	16	75	93	19	73	87
mild symptoms	7	9	13	10	4	6	31	15	12
moderate symptoms	49	11	2	47	20	1	36	10	0
severe symptoms	29	12	0	27	1	0	14	2	1
**Occlusal state Index**									
	**Hyperdivergent(%)**			**Intermediate(%)**			**Hypodivergent(%)**		
	**T0**	**T1**	**T2**	**T0**	**T1**	**T2**	**T0**	**T1**	**T2**
no occlusal disturbances	13	66	96	17	71	89	14	85	95
moderate occlusal Disturbances	40	23	4	39	10	11	35	9	5
severe occlusal disturbances	47	11	0	44	9	0	51	6	0
**Anamnestic dysfunction Index**									
	**Hyperdivergent(%)**			**Intermediate(%)**			**Hypodivergent(%)**		
	**T0**	**T1**	**T2**	**T0**	**T1**	**T2**	**T0**	**T1**	**T2**
no symptoms	15	84	99	18	88	96	18	81	94
mild symptoms	45	13	1	47	10	4	46	11	6
severe symptoms	40	3	0	35	2	0	36	8	0
